# Bioanalysis Using Surface-Enhanced Raman Spectroscopy: From the Perspective of Single Molecules

**DOI:** 10.1021/acsnano.6c00628

**Published:** 2026-04-28

**Authors:** Xiaobin Yao, Jürgen Popp, Jinqing Huang

**Affiliations:** † Department of Chemistry, 58207 The Hong Kong University of Science and Technology, Clear Water Bay, Kowloon, Hong Kong 999077, China; ‡ Leibniz Institute of Photonic Technology (Leibniz-IPHT), Albert-Einstein-Str. 9, Jena 07745, Germany; § Institute of Physical Chemistry, Friedrich-Schiller University, Helmholtzweg 4, Jena 07743, Germany

**Keywords:** single-molecule, surface-enhanced Raman spectroscopy, bioanalysis, signal intensity fluctuation, sample degradation, spatial resolution, quantitative analysis

## Abstract

Single-molecule surface-enhanced Raman spectroscopy (SM-SERS), with single-molecule sensitivity and fingerprint-like specificity, has become a rising tool for probing the physicochemical properties of biomolecules at a single-molecule level. Notably, SM-SERS is generally achieved by complex methodologies with the assistance of rationally designed substrates and single-molecule techniques, often accompanied by significant technical challenges. Given the persistent issues of reproducibility and reliability, this critical perspective does not aim to provide a comprehensive review but instead progresses from a selective overview of representative SM-SERS methods and features to a detailed discussion of its intrinsic and often overlooked limitations, including the long-existing signal intensity fluctuations (SIFs), sample degradation, and the emerging challenge of big data processing and analysis. It is aimed to provide a conceptual framework that advances both the fundamental understanding and practical application of SM-SERS in future research.

## Graphical abstract

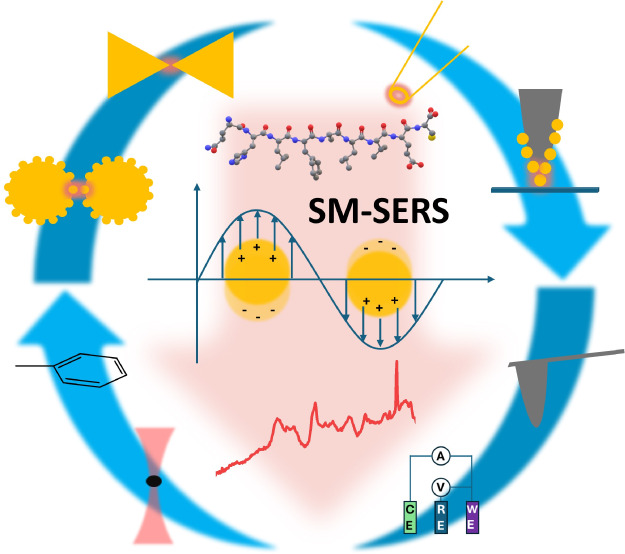

## 1 Introduction

Biomolecules, such as nucleic acids and proteins, as fundamental components of living organisms, play vital roles in various biological processes. To investigate molecular nature, including structures, functions, and roles, particularly at the single-molecule level, is essential for understanding their physiochemical foundations and their relationships to the life processes of living organisms. Meanwhile, these explorations further drive the development of other fields like medicine. Substantial techniques have been employed in the research of biomolecules, where optical techniques show their influences, including microscopy, spectroscopy, and others. Fluorescence microscopy is broadly applied in the investigation of observing, recognizing, and tracking molecules, particularly in *in situ* studies. However, inherent drawbacks, such as photobleaching and phototoxicity of fluorophores, limit its applications in physiochemical investigations at the single-molecule level. In contrast, single-molecule surface-enhanced Raman spectroscopy (SM-SERS) emerges as a more versatile and powerful approach in physiochemical investigations.

Raman spectroscopy analyzes photons inelastically scattered by molecules. As the shifted energies of the photons correspond to vibrational transitions of molecules, it produces unique spectral information like a fingerprint to present the structural information on molecules. However, the intrinsically low Raman cross-section limits the applications of Raman spectroscopy in trace analyses, which is overcome by SERS. In the 1970s, M. Fleischmann and R. P. van Duyne et al. observed and explained the significant Raman signal of pyridine on the surface of a rough silver electrode, respectively, which are regarded as the onset of SERS.^1^ Subsequently, substantial works established the concept that noble metal nanoparticles contribute to the large enhancement of Raman signals, which are proposed to enhance the local electromagnetic field originating from the localized surface plasmon resonance (LSPR) effect. In 1997, the concept of SM-SERS was introduced by two independent groups, where it was mentioned that the Raman signals in SERS experiments can be enhanced up to 10^14^–10^15^ times, which made single-molecule analyses available.^2,3^ Notably, such a substantial enhancement factor (EF) was an early interpretation, and nowadays a more reasonable EF value of ∼10^8^–10^10^ for typical substrates has been broadly accepted.^4^ This was followed 3 years later by the development of tip-enhanced Raman spectroscopy (TERS), a super-resolution SM-SERS technique integrating scanning probe microscopy (SPM), obtaining a super-resolution ability down to ångström-scale resolution under extreme ultrahigh-vacuum conditions, enabling the investigations of small single molecules.^5,6^ Nowadays, SM-SERS continues to attract numerous interest with its fingerprint-like specificity and high sensitivity in the physiochemical investigation of biomolecules.^7^

SERS studies are frequently constrained by complex mechanisms, resulting in conflicting interpretations and ongoing controversy. This is further confounded by long-standing issues of reproducibility and reliability, which lead to a significant underestimation of SERS performance. To better harness SM-SERS for applications in life sciences, this Perspective provides a focused commentary on three critical areas essential for its advancement: the common methods, the core features, and the practical limitations in bioanalysis.

## 2 Common SM-SERS Methods in Bioanalysis

SM-SERS analysis can be broadly categorized into two operational modes based on the manipulation of substrates and/or target molecules. The traditional approach, often termed static SM-SERS, relies on probe molecules or ligands that provide strong Raman signals for the indirect detection of analytes. In contrast, dynamic SM-SERS is an emerging methodology that enables the direct or indirect detection of single molecules through techniques that allow for the active manipulation of either the target molecules or the SERS substrate itself.

### 2.1 Static SM-SERS

SERS substrates can be fabricated by diverse chemical and physical methods. Colloid-based substrates are the most common chemical-reduction method due to their fast and convenient preparation. Typically, silver nitrate or chloroauric acid is reduced by citrate to prepare silver or gold colloids, respectively. However, native colloidal substrates are generally unsuitable for single-molecule detection due to the random distribution of nanoparticles. To solve this issue, colloids are often functionalized with high Raman cross-section molecular reporters, which are broadly applied in immunoassays.^8,9^ In this approach, the nanoparticles are equipped with multiple components, including a Raman reporter for detection, a specific linker for functionalization and selectivity, and a protective shell for stability and contamination protection.^10,11^ For example, Li et al. presented a digital SERS immunoassay using core–shell Au@Ag–Au nanotags for Interleukin-6 (IL-6) detection, where two analytical methods were proposed to validate single-molecule events following a Poisson distribution and to quantify protein biomarkers over a broad linear dynamic range, respectively (Figure 1a).^12^ With their method, a limit of detection (LOD) of 12.4 fg/mL and recoveries from 92.4% to 105.3% in the quantification of IL-6 were achieved, respectively, which is suitable for clinical applications. This approach enables static SM-SERS in detecting and analyzing small molecules from a complex environment. While it can be easily conducted on a conventional Raman setup, complex experimental design and sophisticated substrate engineering are often required, especially for the selection and connection of Raman reporters.

**Figure 1:**
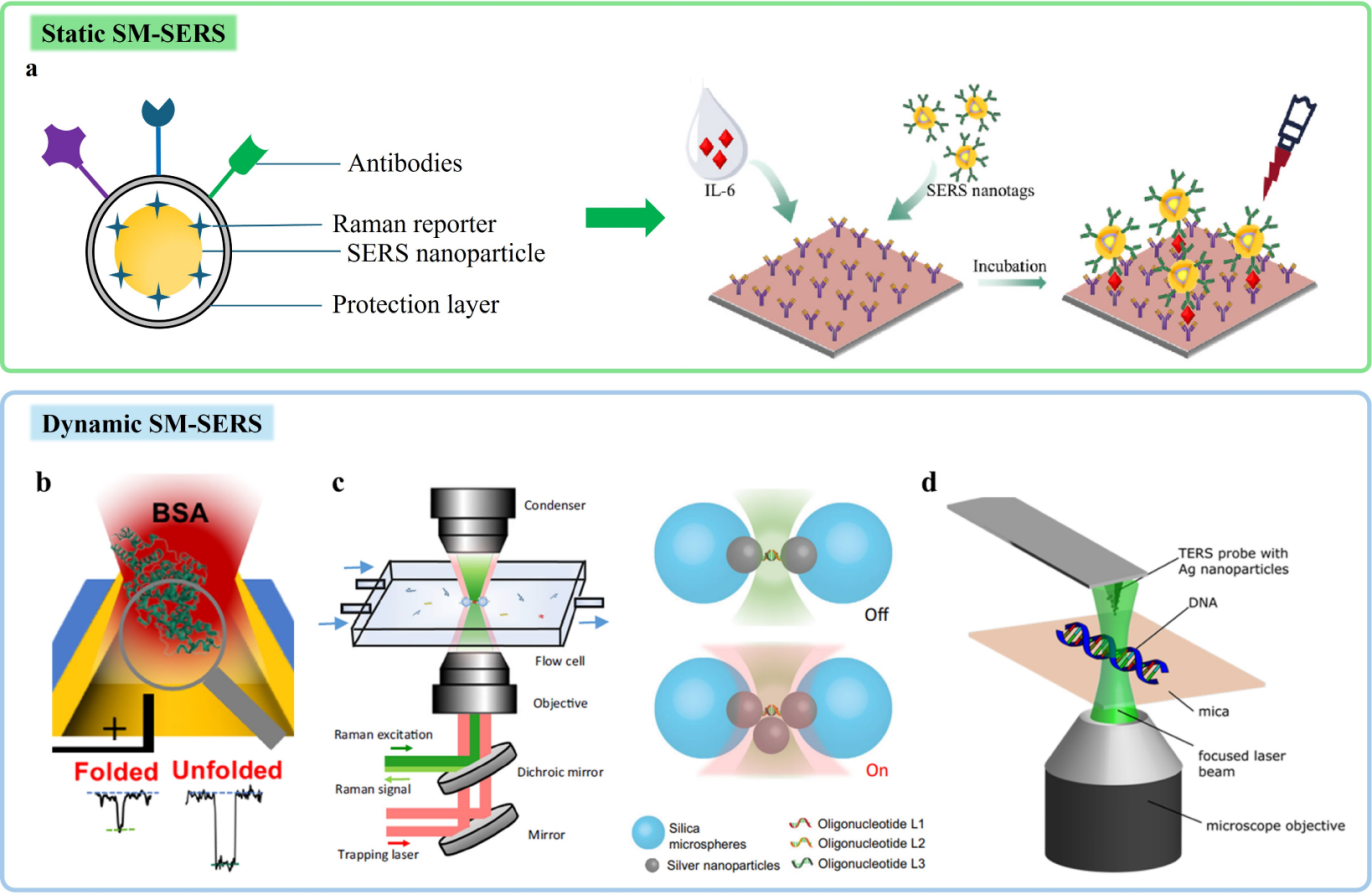
**a.** Schematic illustration of static SM-SERS for immunoassay with the common synthesis process of SERS nanoparticles labeled with Raman reporters. Adapted with permission from ref 12. Copyright 2024 Elsevier B.V. **b–d.** Schematic illustration of dynamic SM-SERS. **(b)** Schematic illustration of a gold plasmonic nanopore for monitoring the unfolding of single-molecule bovine serum albumin (BSA). Adapted with permission from ref 18. Copyright 2025 American Chemical Society. **(c)** Schematic illustration of the OT-SERS setup and different configurations. Adapted with permission under Creative Commons CC-BY 4.0 licenses from ref 19 and ref 20. Copyright 2021 The Authors and published by Springer Nature, and Copyright 2023 The Authors and published by Springer Nature, respectively. **(d)** Sketch of the experimental TERS setup for doxorubicin (DOX)-DNA analysis. Adapted with permission under a Creative Commons CC-BY 4.0 license from ref 23. Copyright 2024 The Authors and published by the American Chemical Society.

### 2.2 Dynamic SM-SERS

In bioanalysis, tracking the structural inhomogeneity and even the dynamic changes of molecules helps in understanding their behavior and functions in the life process. However, static SM-SERS only provides spectral information on analytes at a fixed site and status. To detect component/structural differences or even monitor molecular interactions of analytes, dynamic SM-SERS shows its distinct advantages.^13^ This approach is founded on the active manipulation of either the target molecules or the SERS substrate itself, enabling truly dynamic analysis at the single-molecule level.

Dynamic SM-SERS encompasses several advanced techniques that enable the manipulation and analysis of single molecules. Electrochemical SERS (EC-SERS) is a combination of an electrochemical station and SERS, which can be traced back to the first SERS experiment by Fleischmann et al. In such a technique, the conductive SERS substrate plays the role of an electrode. By tuning the input potential, chemical reactions of adsorbates on the substrate are initiated and monitored, during which real-time spectral information can be acquired. With EC-SERS, more promising research can be realized, such as controllable adsorption/desorption, redox reactions, simulation of biological processes, biosensing, *etc*.^14^ Nowadays, nanopore techniques have emerged to be a powerful tool for single molecule confinement and analysis within nanoscale. While renowned for nucleic acid sequencing, nanopores are increasingly adapted for SM-SERS.^15–17^ By appropriately coating SERS materials like Ag or Au on the inner wall or the entrance of the nanopore, micronano hotspots are created for single-molecule analysis. Notably, to drive the movement of molecules passing through the nanopores, EC stations are often integrated. For example, a gold plasmonic nanopore was employed to monitor the unfolding of single-molecule bovine serum albumin (BSA), where the gradual collapse of the BSA structure induced by high bias voltages was demonstrated through an increase in the fraction of current blockade, and SERS spectra provided structural evidence for protein unfolding (Figure 1b).^18^ In contrast, OT-SERS, a combination of optical tweezers (OT) and SERS, allows simultaneous object manipulation and spectral acquisition in liquid environment with precise control of bead positions.^19–21^ For instance, Fu et al. utilized optical plasmonic trapping to construct a dynamic nanocavity, achieving high-throughput single-molecule characterizations of human Islet Amyloid Polypeptide in aqueous environments, which provided profound mechanistic insight into the pH-regulated amyloidogenesis and introduced an alternative approach for investigating complex biological processes at the single-molecule level (Figure 1c).^20^ However, a significant challenge is the thermal effect generated by the trapping laser (often tens of milliwatts in the infrared), which can be exponentially amplified in the presence of plasmonic nanostructures and may degrade sensitive analytes. Instead of manipulating analytes, TERS realizes SM-SERS by controlling the movement of the integrated SPM, where atomic force microscopy (AFM) and scanning tunneling microscopy (STM) are primarily used, i.e., AFM-TERS and STM-TERS, respectively.^22^ With the confinement of the electromagnetic field at the tip apex of the probe, TERS breaks the optical diffraction limit, achieving nanoscale spatial resolution. Nowadays, a prominent application of TERS is the analysis of DNA molecules with a resolution down to molecular levels.^23,24^ For example, AFM-TERS was applied to identify and localize intercalated doxorubicin (DOX) in DNA, indicating a preferred intercalation of DOX molecules between the guanine and cytosine bases, which provides great potential for qualitative and quantitative evaluation of the potential therapeutic effects of DNA-targeting drugs (Figure 1d).

Apparently, SM-SERS uniquely reveals molecular heterogeneity that is completely invisible to ensemble SERS measurements, where signals of bulk analytes are averaged, and allows the detection of transient conformations, rare structural intermediates, and distinct molecular subpopulations. Moreover, rich variants of SM-SERS techniques enable true real-time observation of structural transitions, reactions, and binding processes at the single-molecule level. Critically, SM-SERS, without fluorophore labeling or invasive manipulations, has the important advantage that such insights can be obtained under conditions much closer to the native biochemical environment than many fluorescence- or force-based single-molecule methods typically allow.

## 3 Features of SM-SERS

Based on the detection sensitivity, SM-SERS can be primarily distinguished from ensemble SERS, which resolves single molecules and trace analytes, respectively. In other words, ensemble SERS acquires bulk properties, while SM-SERS has a true capacity for single-molecule analysis. As highlighted in the above-mentioned studies, typically, SM-SERS relies on single-molecule techniques, very often with a tailored configuration of setup and/or substrate engineering. We can, therefore, roughly separate SM-SERS into two principal components: substrates and combination techniques. To achieve the analysis of single molecules, nanostructures are commonly engineered to ensure good sensitivity for single-molecule detection, such as nanojunctions, bowtie structures, nanopores, and nanogaps (Figure 2a). By confining the plasmonic structures within the nanoscale, the background signals can be well suppressed, which makes the signals of single molecules more reliable. In comparison, combination techniques in SM-SERS, for instance, probe labels, OT, EC, and SPM, are leveraged for molecular or instrumental manipulation, enhancing the reproducibility and control of single-molecule analysis (Figure 2b).

**Figure 2:**
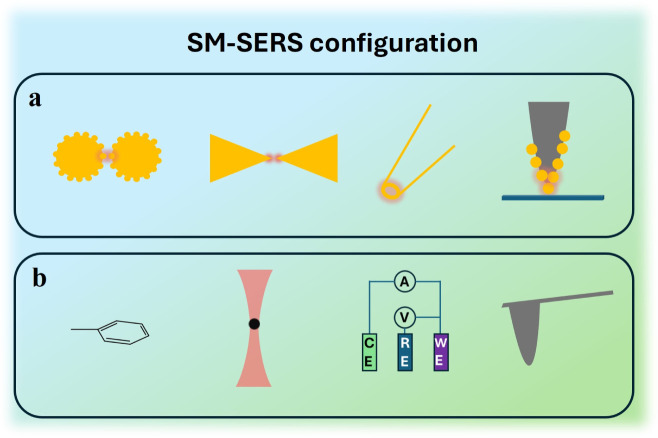
Schematic illustration of some selected SM-SERS. **a**. Various substrates used in SM-SERS, including nanojunction, bowtie, nanopore, and nanogap. **b**. Some common combined techniques for biological SM-SERS, including probe label, OT, EC, and SPM.

Among the SM-SERS methods, a good EF is essentially one of the prerequisites for single-molecule analysis. To realize single-molecule detection, the comprehensive cross-section of a molecule is suggested to ∼10^–19^–10^–20^ cm^2^ sr^–1^ while the intrinsic Raman cross-section of molecules is usually in the range of ∼10^–27^-10^–30^ cm^2^ sr^–1^.^25^ Consequently, an EF of at least ∼10^8^–10^10^ is demanded for a reliable SM-SERS analysis, which can be readily realized by most noble metal substrates. However, we would like to define such an EF as apparent EF (EF_ap_) because it is affected by many experimental variables, such as the properties of molecules and SERS material, *etc*. Additionally, a high specific surface area of a SERS material will increase the adsorption of analytes, confounding the signals of SERS with conventional Raman signals. Therefore, the reported EF should be understood as a specific value for a particular molecule from a defined SERS experiment.

Fundamentally, SM-SERS relies on the nanoconfinement of electromagnetic or chemical enhancement to extremely small volumes, and such confinement is generally achievable with deliberately engineered fabrication of substrates. For example, fine control of the evaporation and deposition procedures is crucial for fabricating TERS tips to ensure good efficiency and reproducibility. In addition, the characteristic stochastic behavior of single-molecule spectra, i.e., signal intensity fluctuations (SIFs), serves as a defining signature that distinguishes SM-SERS from ensemble SERS, while simultaneously introducing significant experimental complexity (Table 1).

**Table 1:** Comparison of Conventional Raman, Ensemble SERS, and SM-SERS

Features	Conventional Raman	Ensemble SERS	SM-SERS
Sensitivity	∼mM-M	<mM	Single molecule
Signal origin	Bulk sample average	Bulk sample average	Single molecule
Substrate requirement	No	SERS materials	Special engineering design
EF	N/A	>10^8^	>10^8^
Laser power	>mW	∼μW-mW	∼μW-mW
Acquisition time	Ten of seconds	Seconds	Seconds
Signal stability	Stable	Stable	SIFs
Signal reliability	Reliable	Degradation	Degradation
Spatial resolution	low	low	Low to high

## 4 Current Bottlenecks toward Robust Bioanalytical SM-SERS

SM-SERS offers a powerful and promising approach to single-molecule analysis, yet its practical application is complicated by several inherent limitations. Among these, SIFs and sample degradation frequently undermine the signal stability and reliability. Furthermore, the technique faces a fundamental constraint: although it achieves single-molecule sensitivity, the spatial resolution of most SM-SERS techniques is diffraction-limited. Additionally, quantitative analysis remains difficult due to the inherently stochastic and noisy spectral data.

### 4.1 Uncontrollable SIFs

SIF is a phenomenon where spectral signals appear or disappear stochastically during measurements. To some extent, it is regarded as a hallmark of SM-SERS due to the trace number of molecules detected.^26^ However, SIFs highly compromise signal stability and interfere with the qualitative and quantitative analysis of SERS. Roughly, the reasons relate to either the substrate or the analyte molecules.

In SERS experiments, the regions with highly intense electromagnetic fields, known as hotspots, are critical for achieving high enhancement and, then, high sensitivity. These hotspots can be nanojunctions, nanocavities, nanoprotrusions, *etc*.^27^ Their roles in SM-SERS experiments have been extensively studied by both computational simulations and experiments. Basically, hotspots are affected by the structures, aggregation states, and properties of nanoparticles, which cause heterogeneous electromagnetic fields and then affect the measurements, leading to SIFs, which are also called SERS blinking. To avoid confusion with fluorescence blinking, the term SIFs is used here. In an SM-SERS measurement, the influence of SIFs is further magnified, shown as spatiotemporal variation, which decreases the reproducibility and reliability of SM-SERS.^28^ Beyond the topic of this perspective, we will not discuss more details about the mechanism of SIFs. It is noted that SIFs can be alleviated by controlling the hotspot arrangement, shortening combination time, decreasing experimental temperature, *etc*. Considering multiplex analysis, etching, and lithography may provide promising solutions for fabricating large-area substrates with uniform and periodic nanostructures, where hotspots at each pitch could be highly similar. This approach can clearly improve the reliability of SM-SERS (Figure 3a).^29,30^

**Figure 3:**
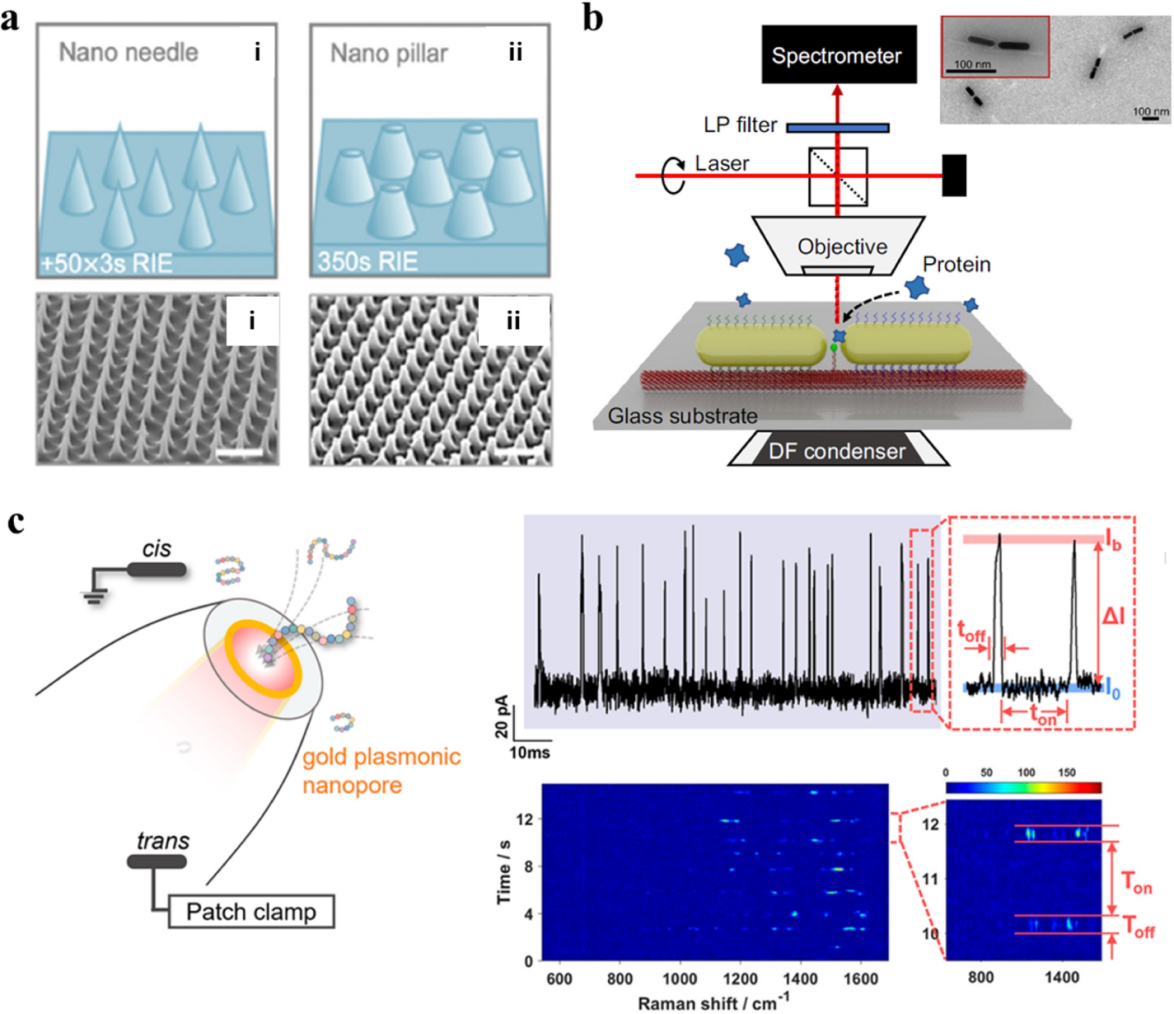
**a**. Repeated patterns for SERS. Adapted with permission from ref 30. Copyright 2024 Wiley-VCH GmbH. **b**. Schematic diagram of a DNA origami-assisted nanorod dimer. Adapted with permission under a Creative Commons CC-BY 4.0 license from ref 34. Copyright 2023 The Authors and published by Springer Nature. **c**. Schematic diagram of the gold plasmonic nanopore for ion current and SM-SERS spectra measurements. Adapted with permission from ref 37. Copyright 2024 American Chemical Society.

With the fine design of SERS substrates, the inhomogeneity of hotspots is expected to be mitigated by reducing the density of hotspots or creating highly uniform structures. However, SIFs are also driven by intrinsic molecular movements (Brownian and thermal), which are difficult to entirely avoid. Currently, several strategies have been proposed to confine single molecules to a small scale, which are expected to alleviate molecular movements. Among these, DNA origami has emerged as a useful tool. It is an artificial nanostructure made by single-stranded DNA and can be served as a programmable scaffold to position two nanoparticles with a controlled gap of just a few nanometers.^31^ With subsequent functionalization of the structure, it can achieve high selectivity and catch specific analytes. Benefitting from the flexible design of structure and gap, various DNA origamis have been reported for single-molecule detection, from simple 2D to intricate 3D structures. For example, a DNA origami scaffold for tip-to-tip alignment of gold nanorods with an average gap size of 8 nm was reported, enabling single-protein SERS spectroscopy with subsecond integration times (Figure 3b).^32–34^ With a tunable interparticle gap and targeted immobilization of analytes, origami-assisted SERS substrates facilitate single-molecule measurements with various dimensions. Moreover, as the nanoparticles are highly confined, this results in more predictable and uniform electromagnetic enhancement, thereby improving signal control. Notably, the main flaw of this strategy is the fabrication procedure of the DNA origami substrate, which is really complex and time-consuming.

In contrast to the immobilization of analytes on static scaffolds like DNA origami, a nanopore-based SERS platform provides a dynamic and promising strategy in SM-SERS, which confines analytes within nanometer-sized pores.^35,36^ By precisely controlling the movement of molecules, individual molecules pass through the nanopore sequentially, which enables true single-molecule detection. For example, an innovative work designed a gold plasmonic nanopore that enabled the high-throughput acquisition of SERS spectra at the single-molecule level by electrically driving analytes into hotspots in detecting and distinguishing not only peptides but also their single-point mutants (Figure 3c).^37^ This nanopore-SERS platform effectively characterized the effects of single-amino-acid substitutions on both the intramolecular interactions and the peptide conformations. Moreover, machine learning was also claimed to overcome SIFs by extracting spectral features of the whole dataset.^38^ Besides, some studies also claimed internal standard methods in improving/solving SIFs issues.^39,40^ Referring to the above-mentioned cases, in our opinion, SIFs cannot be completely eliminated, but they can be mitigated by fine engineering design of substrates and precise control of molecular position and movement.

### 4.2 Unpredicted Sample Degradation

Beyond the substrate issues, a more serious, undesirable, and uncontrollable side effect in SERS is sample degradation induced by plasmonic catalysis. LSPR is not a permanent process and is accompanied by the formation of plentiful electron-holes (also called hot carriers) upon plasmon decay (Figure 4a).^41,42^ These energetic hot carriers are active enough to catalyze the degradation of analytes. Concurrently, thermal dissipation due to the relaxation of hot carriers may also destroy analytes. Even though the complete mechanism of sample degradation is not fully understood, it is widely attributed to a combination of plasmon-driven photocatalysis and thermal catalysis. It is known that biomolecules are extremely fragile and often suffer sample degradation. When a sample is degraded, transient new bands will start to appear and disappear randomly until the sample is completely degraded two broad and strong carbonaceous bands often appear at ∼1350 and ∼1570 cm^–1^ which obscure the original spectrum.^43^ Consequently, critical molecular information will be lost or buried inside the carbonaceous bands, which is one of the main reasons for low reproducibility and reliability in SERS experiments. When this happens in SM-SERS, considering the trace number of analytes, it can be imagined that the situation will be even much worse, as the loss of a single molecule may invalidate the entire measurement.

**Figure 4:**
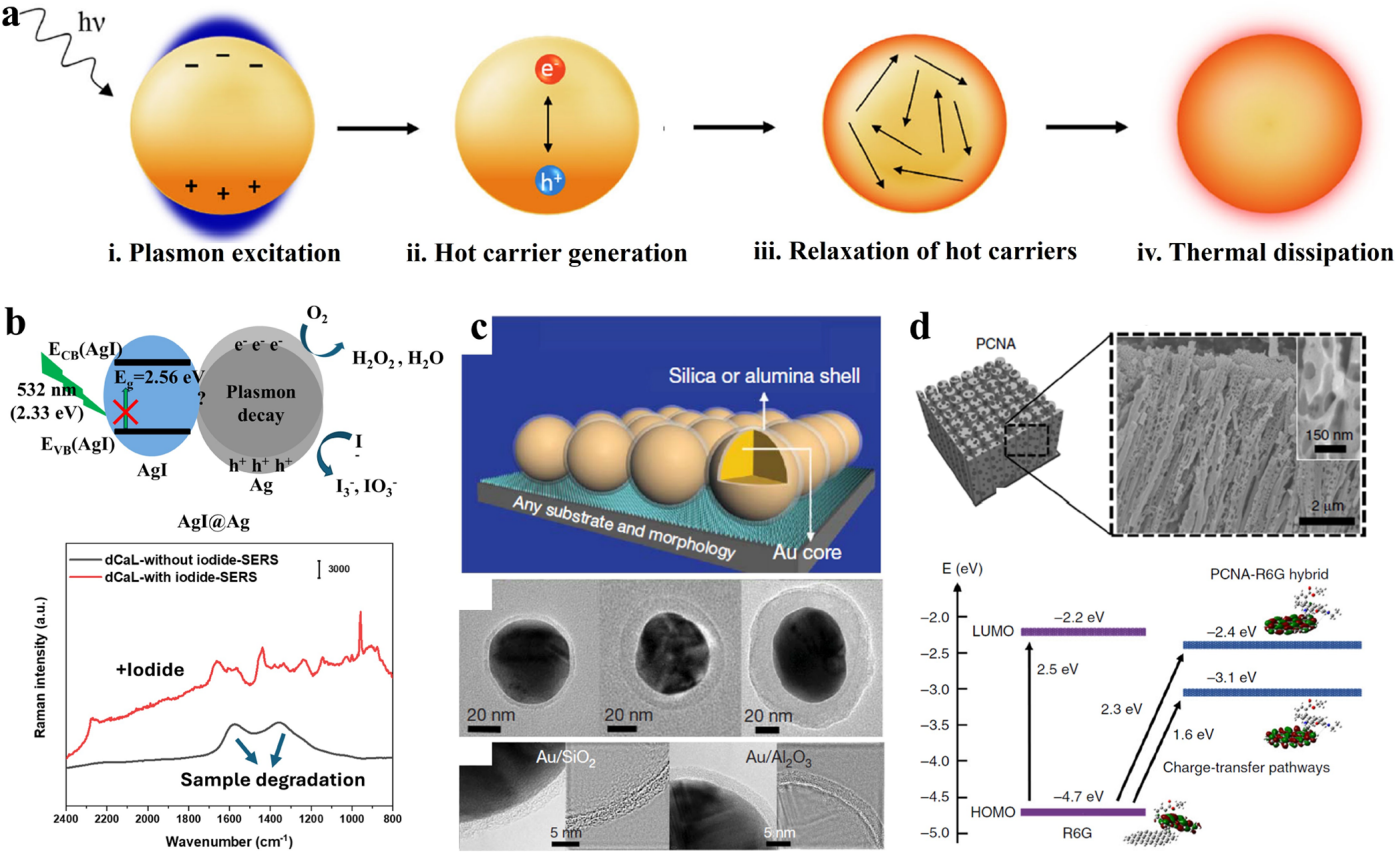
**a**. Schematic diagram of plasmon decay. Adapted with permission under a Creative Commons CC-BY 4.0 license from ref 42. Copyright 2021 The Authors and published by Springer Nature. **b.** Schematic diagram and SERS spectra of targeted suppression of peptide degradation in SERS measurements using iodide ions. Adapted with permission from ref 43. Copyright 2022 Wiley-VCH GmbH. **c**. Schematic illustration and SEM images of SHINERS. Adapted with permission from ref 44. Copyright 2010 Macmillan Publishers Limited. **d**. Sketch and SEM images of PCNA and an energy level diagram showing the molecular orbitals of the model molecule R6G on a carbon sheet. Adapted with permission under a Creative Commons CC-BY 4.0 license from ref 45. Copyright 2020 The Authors and published by Springer Nature.

To mitigate or even circumvent the influence of plasmonic catalysis, several strategies have been noted. Considering catalysis induced by plasmonic electron-holes, it would be a good approach to chemically quench these hot carriers. In our recent study, it was found that iodide ions can well suppress sample degradation of a fungal peptide in SERS experiments (Figure 4b).^43^ It is proposed that iodide ions deplete hot holes due to their smaller redox potentials, which suppresses sample degradation with a chemical method based on the concept of competitive protection. While this method is efficient and convenient, its generality across different systems remains to be verified. Additionally, the interaction between the iodide ions and substrates requires further investigation. In contrast, a physical strategy by blocking the direct contact between analytes and substrates presents another good way. Shell-isolated nanoparticle-enhanced Raman spectroscopy (SHINERS, Figure 4c) is thought to be a useful method; it involved encapsulating a dense inert layer outside the plasmonic nanoparticles, where studies on yeast cells and citrus fruits with pesticide residues illustrate that the SHINERS method significantly expands the flexibility of SERS for useful applications in multiple fields, such as materials and life sciences.^44^ However, two critical aspects must be noted in this technique. First, the basic idea is to screen hot carriers and contaminants from the surface, which means a pinhole-free coating of an inert layer is crucial. Second, as the electromagnetic field decreases exponentially with the distance from the surface, the thickness of the shell must be well optimized.

Given that plasmon decay comes from metal nanoparticles, the removal of metal nanoparticles should also be considered an alternative approach. It is well-known that the enhancement effect of SERS originates from the LSPR and the chemical effect. Even though the LSPR effect dominates the enhancement, the chemical effect can be optimized to a significant level, making them suitable for SERS experiments. As a result, a new group of SERS substrates made by nonplasmonic materials has been developed, and such a technique is called nonplasmonic SERS. A possible mechanism has been proposed, suggesting that the charge transfer between the substrates enhances the local electromagnetic field and enlarges the molecular polarizability, thereby amplifying the Raman signals. Goda’s group presented a method to fabricate porous carbon nanowire arrays (PCNA) as a nonplasmonic SERS substrate, where it is suggested that this effect is due to the strong broadband charge-transfer resonance and the large surface area of the PCNA substrate (Figure 4d).^45^ Besides PCNA, many nonplasmonic materials like carbon and ZnO have been demonstrated to show large SERS activities, which have been applied in bioanalysis.^46^ Remarkably, such a strategy highly depends on orbital coupling, often resulting in high molecular selectivity. Moreover, to compensate for the loss of LSPR, porous materials are often designed to increase the adsorption of analytes, thereby improving the EF_ap_. Nowadays, the field of nonplasmonic SERS is rapidly expanding, with a growing number of innovative substrates being reported.^47,48^

Of the strategies discussed above, both the iodide and SHINERS methods preserve LSPR, while nonplasmonic SERS solely relies on chemical enhancement, which is thought to be a solution for suppressing sample degradation in SM-SERS. There is no doubt that the rapid development of material science brings gigantic possibilities to SERS. However, we worry that the pursuit of a powerful SERS substrate has outpaced the establishment of reliable and reproducible SERS protocols, which leads to debates about the performance of SERS. After half a century, we have to accept the truth that there is still no standard SERS substrate applied. We, therefore, appeal to more researchers to renew their focus and invest their efforts in developing SERS techniques and frameworks that prioritize standardized, reliable analysis.

### 4.3 Low Spatial Resolution

Importantly, it must be noted that single-molecule detection does not equate to high-resolution detection. Essentially, SERS itself is optically diffraction-limited, where the best spatial resolution of SERS is ∼200 nm. With a combination of other supersolution techniques, super-resolution SERS has been realized. For example, by combining stochastic optical reconstruction microscopy (STORM), SERS-STORM has been claimed to own a single-molecule performance with a super-resolution down to ∼10 nm by resolving SERS hotspots (algorithm methods), whose potential is yet not well explored.^49,50^ In contrast, TERS breaks the diffraction limit in another approach (physiochemical methods) by combining a scanning probe microscope with a SERS platform, such as STM-TERS and AFM-TERS. The core principle of TERS is to transfer noble nanoparticles from a large plane to the pinnacle of a sharp tip. In this way, the LSPR effect is confined to the nanoscale end of the tip. Under such a design, the enhanced electromagnetic field is comparable to the size of the tip, and thus, the resolution of TERS can easily reach tens of nanometers. It has been reported that the extreme resolution can even reach ångström scales.^6,51^ The advent of TERS brings the Raman technique into a genuine nanoscale regime and quickly becomes an invaluable tool for bioanalysis, ranging from small molecules to cells.

Remarkably, TERS is well-suited for investigating linear molecules, such as nucleic acids and protein fibrils. A very representative work uses AFM-assisted TERS in the exploration of nucleic acid sequencing. In 2008, TERS was first used in the detection of RNA.^52^ It was demonstrated that TERS can detect signals from a single RNA strand well (Figure 5a). This work indeed provides inspiration for applying TERS in the sequencing field. Due to the structural differences of nucleotides, it has been verified that TERS is able to distinguish them with different spectral information at single-base resolution.^53^ In contrast to squeezing the spatial resolution of TERS in sequencing, it has been extensively applied in structural analysis of molecules like amyloid fibrils.^54,55^ It is noted that all of these early studies were finished on dry samples. The stacking between nucleobases or amino acids clearly interferes with the reading of spectral information. In contrast to experiments under dry conditions, investigations of analytes under physiological conditions are more attractive to bioresearchers. Commonly, an extra adhesive layer is coated between the tip and nanoparticles by physical vapor deposition (PVD) or atomic layer deposition (ALD) methods to prolong the lifetime of the TERS tip in liquid.^56,57^ For example, Lipiec et al. presented their liquid TERS measurements for investigating the secondary structure of amyloid-β aggregates using a Ti–Au coated tip.^58^ However, constrained by intrinsic flaws such as the short tip lifetime in liquid, limited works have been completed using liquid measurements. Besides AFM-TERS, STM-TERS also plays an important role in single-molecule analysis. Recently, a subnanometer-resolved low-temperature STM-TERS method was developed to resolve, in real space, individual nucleobases and their sequence structures within an artificially designed short, single-stranded DNA molecule.^24^ The TERS mapping over individual nucleobases provides additional structural information about the molecular configurations and even the locations of functional groups, which offers unprecedented insight into biomolecular structure and interaction sites (Figure 5b).

**Figure 5:**
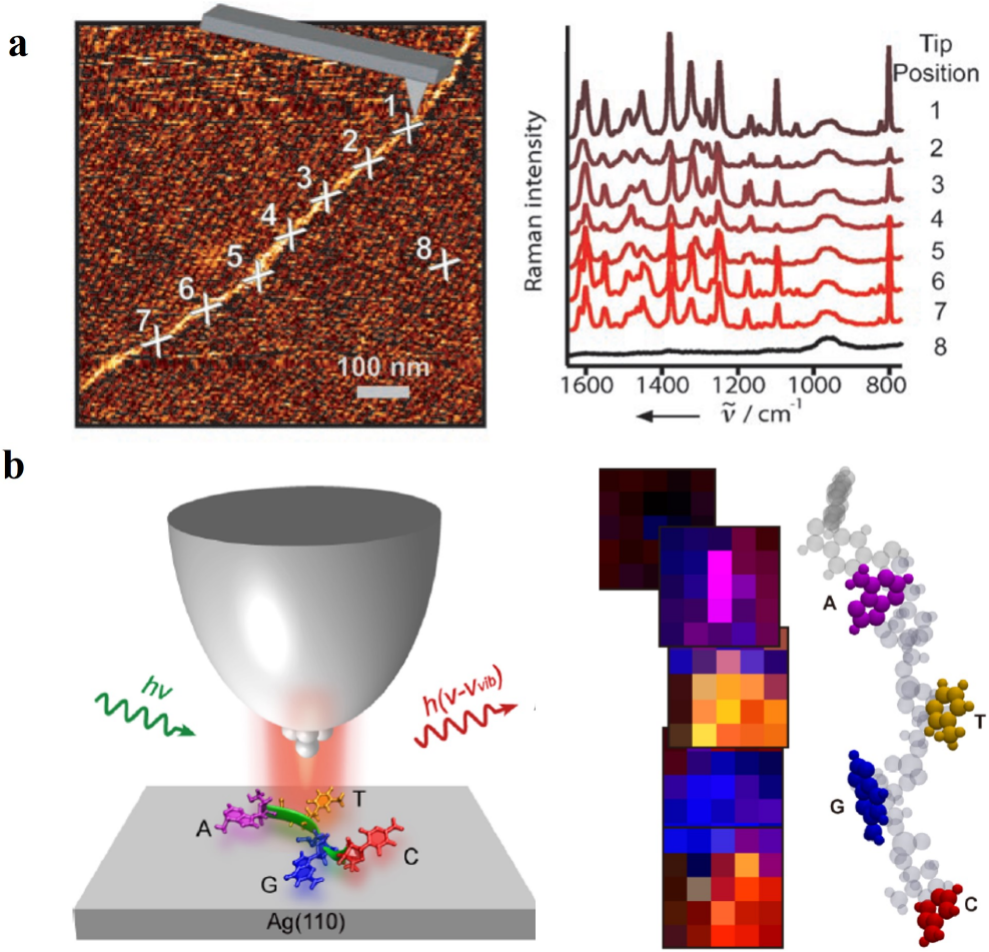
**a.** Schematic illustration of the AFM-assisted TERS measurement along an RNA molecule. Adapted with permission from ref 52. Copyright 2008 WILEY-VCH Verlag GmbH & Co. KGaA, Weinheim. **b.** Schematic diagram of TERS measurements for a single-stranded DNA molecule. Adapted with permission from ref 24. Copyright 2024 American Chemical Society.

Indeed, TERS is a very promising high-resolution optical technique in bioanalysis at the surface and interface. Its combination of high sensitivity, specificity, and intrinsic label-free nature makes it ideally suited for the physiochemical investigation of small molecules within the nanoscale. However, the undeniable truth is that the application of TERS in biological sciences remains limited, with relatively few groups utilizing TERS setups. Some technical challenges may be responsible for this gap. First, the preparation of high-quality tips, typically requiring an ultrahigh vacuum condition, is difficult and time-consuming. Second, measurements in liquid, especially under physiological conditions, highly shorten the lifetime of the TERS tip, while these are highly valued by bioresearchers, as investigations of the native statuses of analytes provide more valuable information about their roles in living processes. Additionally, while direct sequencing using TERS has been verified to be completely feasible, most of the case studies were conducted with dry samples. In our opinion, further technical developments of TERS must focus on the above-mentioned barriers. A simpler method to fabricate more robust tips, particularly a liquid-resistant tip for use under physiological conditions, will make TERS more accessible and practical in bioresearch. Despite the limitations, considering the performance of single-molecule analysis within the nanoscale, we still believe that TERS is an ideal single-molecule technique in biophysical and physiochemical analysis, which is anticipated for broader applications in life sciences in the coming decades.

### 4.4 Challenging Quantitative Analysis

For an analytical technique, quantitative evaluation is fundamental and essential. A common quantitative method in SERS is to create a fitting curve correlating Raman intensity with concentration, while it introduces a high uncertainty at the single-molecule level due to inherent signal variation. As introduced previously, the heterogeneity of hotspots and molecular movements causes apparent intensity fluctuations of a single molecule spatiotemporally, which directly undermines the reliability of a quantitative test. Additionally, when a complex background is involved, the accuracy of the quantitative test is further compromised. In a previous study, it was demonstrated that a convolutional neural network (CNN) model applied to bundles of SERS spectra yields a robust, facile method for concentration quantification down to 10 fM using SM detection events.^59^ Nowadays, machine learning has become a good tool in the quantitative analysis of SERS data down to SM levels.^60,61^

Recently, digital quantification by counting signals has provided a more feasible strategy for single-molecule analysis. This concept involves detecting and counting the appearance of targeted signals in every detection, primarily established in fluorescence-based methods for multiplex analysis, which has been well adapted in SM-SERS (termed as digital SERS).^62,63^ For instance, a static SM-SERS approach was reported for real-time single cytokine counting, enabling dynamic tracking of immune toxicities in cancer patients (Figure 6a).^64^ With the assistance of various cytokine biomarkers, it has been demonstrated that the digital nanopillar SERS assay achieves both highly specific and highly sensitive cytokine detection down to the atto-molar level. Recently, Bi et al. presented a dynamic SM-SERS method called digital (nano) colloid-enhanced Raman spectroscopy (dCERS), which was introduced to realize reproducible quantification of various target molecules.^65^ From the schematic diagram, colloidal nanoparticles are monodispersed inside a capillary, and signals are acquired from each voxel (Figure 6b). In the digital process, a threshold is set to differentiate valid signals from background noise. When the signals are higher than the threshold, the event is counted as “1”, otherwise, it is counted as “0”. Clearly, this idea is distinct for the detection of ultralow concentration samples, especially single molecules. However, when the concentration is too high, the single-molecule events may decrease, and the overlapped signals will cause the deviation of the response. Therefore, an appropriate single-molecule concentration of target molecules should be predetermined. Besides small molecule detections, digital SERS has also been applied in the quantification of larger biotargets like viruses and yeast cells.^66,67^

**Figure 6:**
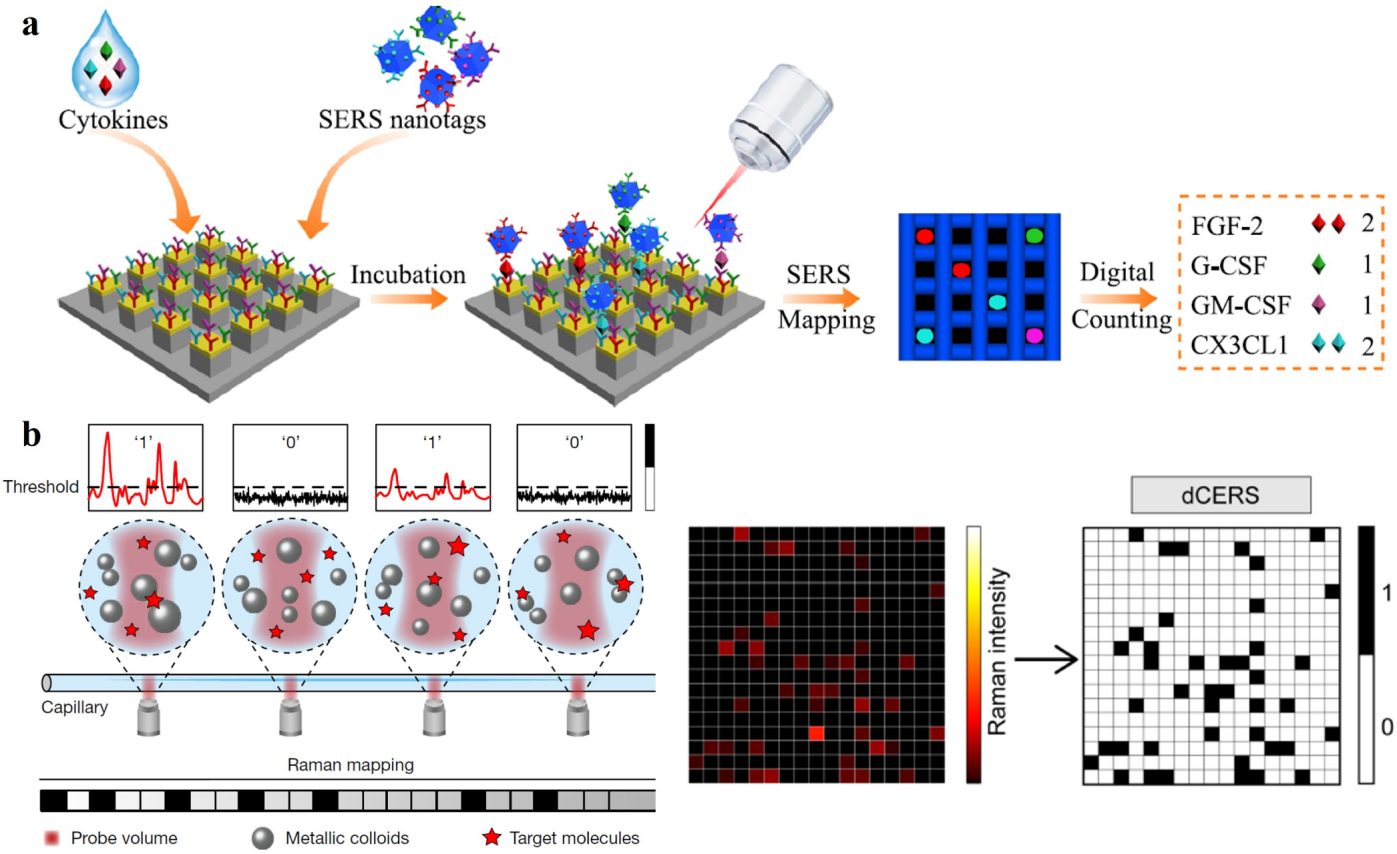
**a.** Schematic illustration of the digital single-molecule nanopillar SERS platform for parallel counting of four types of cytokines. Adapted with permission under a Creative Commons CC-BY 4.0 license from ref 64. Copyright 2021 The Authors and published by Springer Nature. **b.** Schematic diagram of dCERS and intensity/digital map indicating detected target molecules. Adapted with permission from ref 65. Copyright 2024 The Authors under exclusive license to Springer Nature Limited.

From our perspective, digital SERS presents the most applicable strategy in the quantitative analysis of SM-SERS experiments. However, cumbersome preparation and predetermination processes become the largest barrier. First, a preset threshold should be defined, which directly relates to the detection error. For an indirect measurement with Raman reporters, the influence of the threshold should be minor. However, when it becomes involved in a direct measurement of a biomolecule with a low Raman cross-section, a substantial error may be introduced. The setting of the threshold affects not only the false positive rate but also the false negative rate. Due to the influence of hotspots and molecular movement, some genuine information on molecules may not be well detected, making the false negative rate a serious concern . Second, it should be noted that this technique highly relies on experimental conditions, especially the detector response, which may affect the efficacy and accuracy of detection. Third, analytes should have distinguishable signals compared to the background, which requires a good signal-to-noise ratio from experiments. Moreover, the chemical interactions/reactions of molecule–molecule and molecule–substrate must be minimized to avoid potential structural changes in molecules, especially at an ultralow concentration. Furthermore, to increase accuracy, a large enough measurement volume is required to follow the Poisson distribution. Finally, data analysis is another concern about the general application of digital SERS. We suggest exploring the integration of digital SERS with machine learning, which holds promise in signal extraction and big data analysis.

### 4.5 Complicated Data Analysis

While suitable experimental control affects data acquisition, robust data analysis is essential for the presentation of critical information. In SM-SERS, two major challenges make data analysis complicated. First, spectra from similar components are difficult to distinguish. Second, high background noise and SIFs decrease the accuracy of the data analysis. To overcome these issues, machine learning offers good assistance, which has been applied in classification, clustering, and prediction in the research of biomedicine, diagnostics, *and others*.^68–70^ Guo et al. presented a detailed protocol for data analysis of Raman experiments, from experimental design to machine learning (Figure 7a).^71^ In an SM-SERS measurement, the data volume will highly affect the final accuracy of the data analysis. In some cases, millions of data will be acquired. Importantly, data preprocessing is a vital step in machine learning, such as spike removal, and wavenumber and intensity calibration. While baseline correction can remove the influence of fluorescence, it must be noted that some useful information may be subtracted at the same time. Once data preprocessing is finished, a suitable model can be selected for machine learning. The most common machine learning method is linear regression, which is often used to investigate the relationship between variables. Another common tool, principal component analysis, is a type of dimension reduction tool that reduces the number of big data sets to principal components with main information and presents these data in a coordinate system. With the increase in data complexity, various artificial neural networks are often employed in data classification and identification.

**Figure 7:**
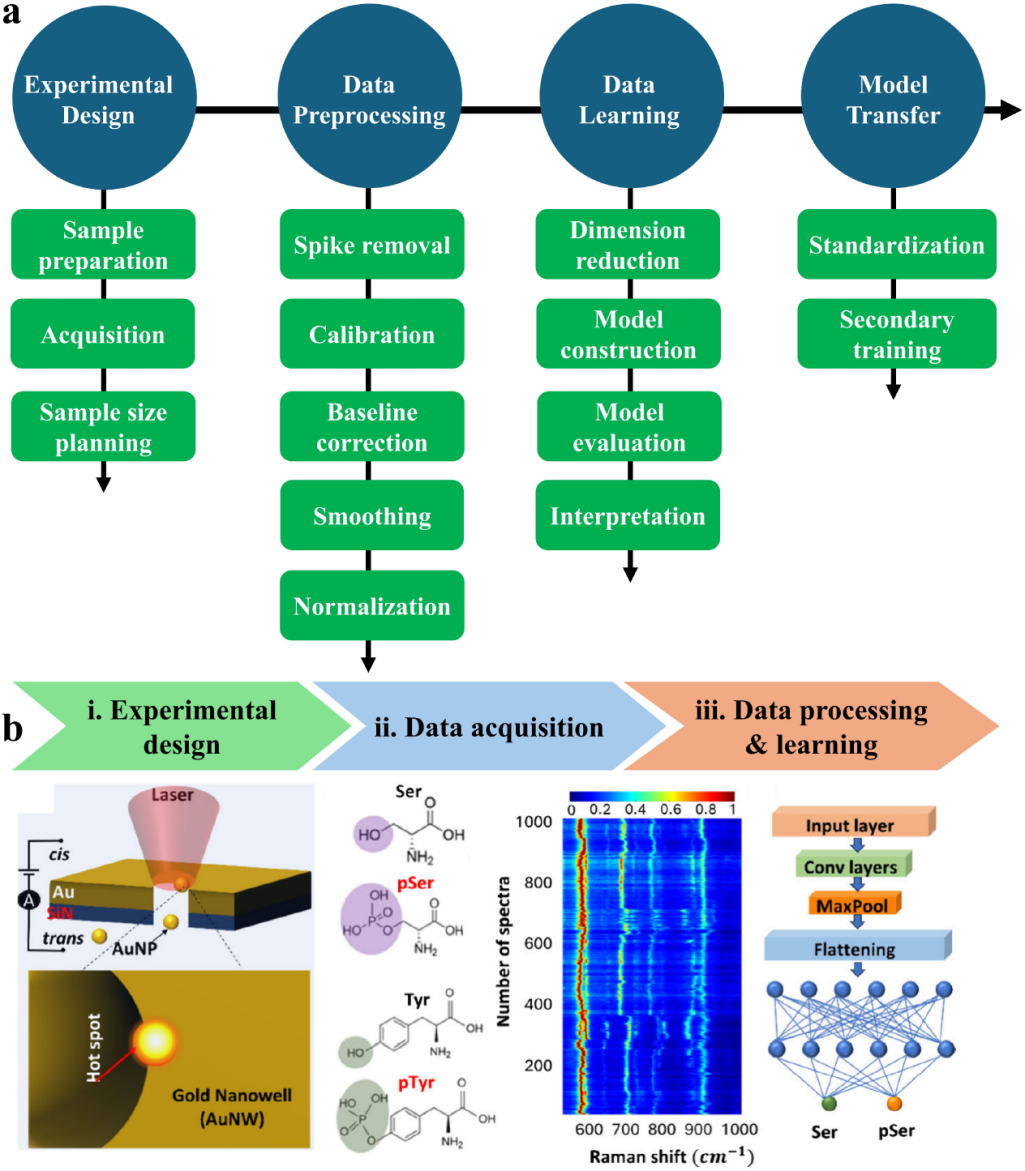
**a**. Working protocol for Raman experiments. Adapted with permission from ref 71. Copyright 2021 The Authors under exclusive license to Springer Nature Limited. **b**. Schematic of the deep learning-assisted SERS method for single post-translational modification (PTM) detection. Adapted with permission under a Creative Commons CC-BY 4.0 license from ref 73. Copyright 2025 The Authors. Published by the American Chemical Society.

Currently, machine learning-supported SERS analysis is rapidly thriving.^38,70,72^ Recently, Yaltaye et al. developed a high-accuracy SM-SERS approach by combining a plasmonic particle-in-pore sensor to collect SM-SERS spectra of phosphorylation at serine and tyrosine, k-means-based clustering for citrate signal removal, and a one-dimensional convolutional neural network (1D-CNN) for phosphorylation identification (Figure 7b).^73^ In this model, the authors used more than 15,000 amino acid spectra for training, validation, testing, and postevaluation. Finally, it was demonstrated that SM-SERS data with submonolayer analyte coverage of the particle surface discriminated the phosphorylation in serine and tyrosine with over 95% and 97% accuracy, respectively. Such an accuracy difference is proposed for the structural differences between amino acids and peptides. This case raises some key considerations about machine learning in SM-SERS. First, data preprocessing will greatly affect the accuracy of machine learning. Moreover, every model should be trained, checked, and reevaluated frequently in real analyses and cannot simply be applied to other different cases. Even though a more complex model means higher accuracy, a larger data volume and a longer learning period are required. However, the time performance in machine learning is not clearly mentioned or discussed, which should also be a key criterion in developing machine learning workflows for SM-SERS. Regardless, the benefit of machine learning in processing complicated data is noticeable, and the development of machine learning is also rapid. We anticipate that more sophisticated and efficient machine learning workflows will be integrated into the study of SM-SERS, enabling more precise analysis.

Although SM-SERS is complicated by several bottlenecks, some promising solutions have emerged. First, signal instability and sample degradation, stemming from hotspot heterogeneity, plasmon-mediated chemistry, and uncontrollable molecular motion, fundamentally challenge the technique’s reliability and reproducibility. These issues can be alleviated or addressed by the assistance of engineering substrates, sample nanoconfinement, and machine learning, among other strategies. Second, the spatial resolution of most SM-SERS techniques remains diffraction-limited depending on the research scenarios, which can be addressed by algorithm-assisted SERS-STORM or SPM-assisted TERS techniques. Third, quantitative analysis is hindered because single-molecule spectra are inherently stochastic and noisy, which has been recently well-solved by digital counting strategies and machine learning. In summary, despite these current challenges, continued development of innovative approaches still offers a promising path toward robust bioanalytical- or even clinical-grade SM-SERS.

## 5 Outlooks

SM-SERS has emerged as a powerful single-molecule analytical technique that offers exceptional sensitivity, molecular specificity, and high spatial resolution. In this Perspective, we outline common methodologies, clarify defining features, and critically discuss persistent limitations of SM-SERS. The conceptual framework, by categorizing SM-SERS into static and dynamic modes, is refined based on the manipulation of substrates and/or target molecules. Fundamentally, a reliable SM-SERS analysis requires an engineered substrate providing a high EF. Furthermore, to address challenges from data acquisition to analysis, notably on the issues of reproducibility and reliability, we interrogate critical technical bottlenecks such as SIFs, sample degradation, spatial resolution constraints, and difficulties in quantification and data analysis, while introducing corresponding potential solutions. Moreover, the construction of a robust and straightforward “standard experiment” will be essential to validate the reliability of SM-SERS, which would require the clear distinction of two spectroscopically different analytes, such as two adjacent amino acids on a peptide chain or free amino acids in a mixture—a capacity that highly depends on the sensitivity and spatial resolution of the SM-SERS techniques. By clarifying these scientific questions, we aim to foster a deeper understanding of SM-SERS. Optimistically, it is anticipated that more advanced and ingenious analytical methods leveraging high sensitivity and super-resolution SM-SERS techniques, such as label-free protein or nucleic acid sequencing, will soon be established and realized. Ultimately, the continued advancement of SM-SERS is expected to significantly accelerate progress in the life sciences by enabling sophisticated and creative single-molecule investigations.

## Abbreviations

SERS: surface-enhanced Raman spectroscopy
SM-SERS: single-molecule surface-enhanced Raman spectroscopy
LSPR: localized surface plasmon resonance
TERS: tip-enhanced Raman spectroscopy
SPM: scanning probe microscopy
EC-SERS: electrochemical surface-enhanced Raman spectroscopy
OT-SERS: optical tweezers surface-enhanced Raman spectroscopy
STM: scanning tunneling microscopy
AFM: atomic force microscopy
EF: enhancement factor
SIF: intensity fluctuation;
SHINERS: shell-isolated nanoparticles-enhanced Raman spectroscopy
PCNA: porous carbon nanowire arrays
dCERS: digital (nano) colloid-enhanced Raman spectroscopy
1D-CNN: one-dimensional convolutional neural network.
